# Are environmental characteristics in the municipal eldercare, more closely associated with frequent short sick leave spells among employees than with total sick leave: a cross-sectional study

**DOI:** 10.1186/1471-2458-13-578

**Published:** 2013-06-13

**Authors:** Christina Malmose Stapelfeldt, Claus Vinther Nielsen, Niels Trolle Andersen, Line Krane, Nils Fleten, Vilhelm Borg, Chris Jensen

**Affiliations:** 1 Section of Social Medicine and Rehabilitation, Department of Public Health, Aarhus University, Aarhus, Denmark; 2 Public Health and Quality Improvement, Central Denmark Region, Aarhus, Denmark; 3 Section of Biostatistics, Department of Public Health, Aarhus University, Aarhus, Denmark; 4 Department of Community Medicine, Faculty of Health Sciences, University of Tromsø, Tromsø, Norway; 5 National Research Centre for the Working Environment, Copenhagen, Denmark; 6 National Centre for Occupational Rehabilitation, Rauland, Norway

**Keywords:** Cross-sectional, Home care services, Psychology, Social, Sick leave, Working environment

## Abstract

**Background:**

It has been suggested that frequent-, short-term sick leave is associated with work environment factors, whereas long-term sick leave is associated mainly with health factors. However, studies of the hypothesis of an association between a poor working environment and frequent short spells of sick leave are few and results are inconsistent. Therefore, we aimed to explore associations between self-reported psychosocial work factors and workplace-registered frequency and length of sick leave in the eldercare sector.

**Methods:**

Employees from the municipal eldercare in Aarhus (N = 2,534) were included. In 2005, they responded to a work environment questionnaire. Sick leave records from 2005 were dichotomised into total sick leave days (0–14 and above 14 days) and into spell patterns (0–2 short, 3–9 short, and mixed spells and 1–3 long spells). Logistic regression models were used to analyse associations; adjusted for age, gender, occupation, and number of spells or sick leave length.

**Results:**

The response rate was 76%; 96% of the respondents were women. Unfavourable mean scores in work pace, demands for hiding emotions, poor quality of leadership and bullying were best indicated by more than 14 sick leave days compared with 0–14 sick leave days. For work pace, the best indicator was a long-term sick leave pattern compared with a non-frequent short-term pattern. A frequent short-term sick leave pattern was a better indicator of emotional demands (1.62; 95% CI: 1.1-2.5) and role conflict (1.50; 95% CI: 1.2-1.9) than a short-term non-frequent pattern.

Age (= < 40 / >40 years) statistically significantly modified the association between the 1–3 long-term sick leave spell pattern and commitment to the workplace compared with the 3–9 frequent short-term pattern.

**Conclusions:**

Total sick leave length and a long-term sick leave spell pattern were just as good or even better indicators of unfavourable work factor scores than a frequent short-term sick leave pattern. Scores in commitment to the workplace and quality of leadership varied with sick leave pattern and age. Thus, different sick leave measures seem to be associated with different work environment factors. Further studies on these associations may inform interventions to improve occupational health care.

## Background

The public sector in general and the municipal health care sector in particular is challenged by high sick leave rates among home-care personnel [1-3]. The sector also reports problems in recruiting and holding on to new employees. Demographic changes marked by a growing size of the population of elderly citizens, expectably in demand of personal care and home care, adds further to this vicious circle [4]. Several models have been developed in an attempt to capture positive and/or negative factors in the working environment explaining adverse effects among employees, i.e. sick leave, turn-over intentions and low engagement. Two models have gained general acceptance in the field of occupational health; effort-reward imbalance model [5], and the demand-control-(support) model [6]. However, these two models may be too static, i.e. the items used do not offer adequate descriptions of important work environment characteristics in all occupational settings [7]. A more recent model (the job demands-resources model) [8] proposes that every organisation has its own unique work environment characterised by demands and resources. It further claims that health may be affected by sustained high job demands and turnover intentions may be a consequence of sustained low job resources [7].

The National Research Centre for the Working Environment (NRCWE) conducted a national survey of the municipal eldercare in Denmark in 2004–2005 [9]; the survey reported a 34% higher level of sick leave among home-care personnel than among administrative employees within the eldercare sector [10]. Numerous demands and resources in the psycho-social and physical working environment along with lifestyle factors were found to be associated with high self-reported sick leave levels [10]. The survey also reported a significantly higher risk of having high (more than four weeks) and moderate (one to four weeks) levels of sick leave among young homecare employees (<40 years). This increased risk was partly explained by the experience of more role conflict, less commitment to the work place and less wellbeing [11].

It has been suggested that frequent short-term sick leave is associated with work environment factors, whereas long-term sick leave is associated mainly with ill health and reduced workability [12,13]. However, few studies have analysed if psychosocial work factors are more strongly associated with frequent short-term sick leave than with long-term sick leave [14]. In studies on health care workers, the total number of sick leave days per year was associated with high psychological demands, high physical workload [15] and low social support [16]. The number of sick leave spells was found to be related to physical and psychological work demands [16-19]. Elstad et al. reported proportionality in the association between the number of perceived stressful work characteristics and the number of spells [20]. Bullying is another factor that is associated with an increased risk of a new spell of sick leave [18]. High levels of decision authority, perceived meaningfulness of work, commitment to the workplace, quality of leadership and a good team climate may protect against sick leave [17,21,22]. The effect of social support on spells of sick leave varies between studies [16,19,23].

Generally, younger age is related to a higher frequency of spells [17,19,20,23,24], and older age is associated with fewer, but longer spells [17,18,24]. Moreover, age seems to modify the associations between work factors and different sick leave measures [25].

Apart from the effect of age, inconsistencies in the reported associations between work factors and sick leave are considerable. This was suggested to be due partly to the different measures of sick leave themselves [12,26]. Hence, most studies analyse length of sick leave and frequency of spells separately, and any independent association between length versus frequency and psychosocial work factors therefore remains unknown. However, in a study where nursing assistants were compared with doctors, the former had a higher risk of having incident sick leave spells of 1–3 days (adjusted for medium and long spells) and sick leave spells of 4–14 days (adjusted for short and long spells) than the latter [27].

The high levels of sick leave in the elderly care indicate that early detection of unfavourable changes in the working environment may be instrumental in maintaining the employees’ wellbeing, productivity and viability within the institution or company. According to the literature, an association between total sick leave length and a poor working environment may be more likely to be due to a frequent short-term sick leave pattern than a non-frequent long-term sick leave pattern [12-14], and even more so in young employees than in older colleagues [11]. Long-term sick leave spells, on the other hand, seem to be more strongly associated with a poor general health than short spells. Whether these assumptions are valid within the elderly care sector has not yet been established.

This study draws on workplace-registered sick leave data to explore if sick leave patterns, i.e. the number of short-term sick leave spells or merely their duration, are associated with self-reported, unfavourable psychosocial work factors. If associations can be identified, the employers’ sick leave register may be a fruitful source for initiating preventive work environment measures.

### Aim

This study aims to explore associations between sick leave patterns and psychosocial work factors in elderly care and thereby enhance the potential for using the employers’ sick leave register to launch interventions aimed at improving the working environment and, hence, the employees’ wellbeing, productivity and viability.

## Methods

A cross-sectional design was chosen because we wanted to use sick leave measures as an indicator of a poor working environment; not to study causal pathways.

We used workplace-registered sick leave data from municipal eldercare workers in Aarhus, Denmark. Questionnaire data on the working environment were collected by the NRCWE, Copenhagen in 2005.

### Study population

**Figure 1:**
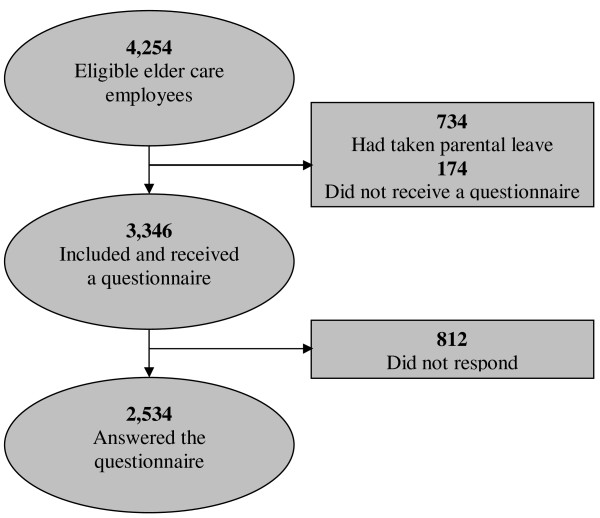
Flowchart of the selection of eldercare employees into the study.

Survey data on psychosocial work factors were merged with workplace-registered sick leave records from 2005. Sick leave from 2005 was chosen because all of the respondents had answered the questionnaire at some point between 18 February and 18 July 2005. Employees were included if they had been employed and had been working throughout 2005 and had received a questionnaire (N = 3,346) (Figure 1).

### Sick leave patterns

Data on absence in 2005 were retrieved from the workplace records, which also contained stated reasons for each absence spell. Only spells related to sick leave were included. The dates of the first and the last day of each sick leave spell were available for each individual. A sick leave spell was counted in calendar days regardless of whether all of these days were work days. Overlapping, consecutive or duplicate sick leave spells were merged into a single spell.

First, sick leave was measured as the total number of sick leave days including all spells ended in 2005. We dichotomised this measure into 0–14 days and 15–581 days [10,11,25,28,29]. In 2005, the public insurance system reimbursed sickness benefits for sick leave spells lasting more than 14 days in conformity with the Danish Sickness Benefit Act [30].

Second, sick leave was categorised into patterns with short, long or a combination of short and long spells. We defined short spells as spells lasting zero to seven calendar days. This definition has also been used in other studies [24,31]. Long spells were defined as spells lasting eight calendar days or more.

Based on the above-mentioned measures, the sick leave patterns were:

Length of sick leave per year: *0*–*14 days* and *more than 14 days*.

Spell patterns: *0*–*2 short spells*, *3*–*9 short spells*, *2*–*13 mixed spells* and *1*–*3 long spells*.

### Psychosocial work environment factors

The Copenhagen Psychosocial Questionnaire (COPSOQ) [32] was used to collect information on perceived work-related demands and resources from the eldercare workers.

The environmental factors suggested to be the most important variables in explaining the self-reported high levels of sick leave among eldercare workers [10,11] were selected from the survey. These were: work pace, emotional demands, demands for hiding emotions, physical work load, influence, meaning of work, commitment to the workplace, role conflict and quality of leadership. These factors were scored on five-point Likert scales (*Always*, *Often*, *Sometimes*, *Seldom*, *Never*/*hardly ever*). The scores were transformed into 0–100 scores. The mean values of the different work environment scales were measured in a representative sample of working Danes in 2004/2005 [33]. In the present study, the selected work environment scales were dichotomised into favourable / unfavourable scores according to these mean scores. This was done for all scales except for physical work load which was dichotomised according to the mean value found in our data. Response categories on bullying and threats of violence were “*at least once a month*”, “*from time to time*” and “*never*”. They were dichotomised into “*at least from time to time*” and “*never*”. Response categories on general health were “*Excellent*”, “*very good*”, “*good*”, “*fair*” and “*poor*”. This variable was dichotomised into “*excellent*-*good*” and “*fair*-*poor*”. Reference scores were not available for bullying, threats of violence and general health.

### Socio-demographics and potential confounders

Age (= < *40*/>*40 years*) and gender were retrieved from the personnel files, whereas occupation (categorised in *administration*, *manager*, *therapist*/*nurse*, *home care* and *non*-*care*) was retrieved from the questionnaire. We used the cut point of 40 years of age so direct comparisons with the results of the analyses carried out by NRCWE (10;11) were possible.

### Statistical analyses

Univariable and multivariable logistic regression models were applied to find associations between total sick leave days/spell patterns and unfavourable scores in work factors and general health.

In the first analysis, using total sick leave days as the independent variable, we adjusted the model for age, gender, occupation and number of spells (*continuous variable ranging from 0*–*13*).

In the second analysis, using spell pattern as the independent variable (0–2 short-term sick leave spells was the reference), we adjusted the models for age, gender, occupation and total sick leave days categorised in four (*0 days*, *1*–*14 days*, *15*–*56 days* and >*57 days*). A Wald-test was performed to establish the overall difference in the odds of having unfavourable work factor scores between the sick leave patterns.

In the third analysis, using spell pattern as the independent variable, we again performed the same adjustments as mentioned in the second analysis. Based on the literature, we expected that unfavourable work factor scores and total sick leave length were more likely to be due to frequency of spells than duration, and that this association would be more pronounced among young employees than among their older colleagues. Long-term spells, on the other hand, were believed to be more strongly associated with poor general health than short-term spells. In this analysis, emphasis was on the odds of having unfavourable work factor scores / general health scores among eldercare workers with many short spells compared with few, but long spells (reference). We also sought to establish whether these associations were stronger among young employees than among older colleagues by including an interaction term between age and spell pattern.

The scale reliability coefficient (Cronbach’s alpha) of work factors used from the COPSOQ was calculated. To establish whether the work factor scores and general health scores were statistically significantly different between total sick leave categories and sick leave patterns, t-test, one-way ANOVA and chi2 was applied.

The significance level was set at p < 0.05. The results are shown as crude and adjusted figures.

STATA version 12.1 was used as statistical software.

Approval (2012-41-1290) for using workplace-registered sick leave records and questionnaire data was obtained from the Danish Data Protection Agency: http://www.datatilsynet.dk/english/.

## Results

The questionnaire was answered by 2,534 employees, which yielded a response rate of 76%. Non-responders were more likely to be young and male than responders (Table 1). Non-responders were also more likely to have a higher number of total sick leave days and fewer and longer spells of sick leave (Table 1).

**Table 1:** Age, gender and sick leave among responders (n = 2,534) and non-responders (n = 812) to the work environment questionnaire

	**Responders**	**Non**-**responders**	**P**-**value**
**Age**, median (min-max)	49.2 (19–68)	47.2 (18–68)	<**0**.**001**
**Female**, n (%)	2,433 (96)	751 (93)	<**0**.**001**
**Total sick leave**, n (%)			<**0**.**001**
0-14 days	1,909 (75)	549 (68)	
More than 14 days	625 (25)	263 (32)	
**Spell patterns**, n (%)			<**0**.**001**
0-2 short spells	1,378 (54)	380 (47)	
3-9 short spells	490 (19)	162 (20)	
2-13 mixed spells	515 (20)	183 (23)	
1-3 long spells	151 (6)	87 (11)	
**Total number of spells**,			
Median (min-max)	2 (0–13)	2 (0–11)	**0**.**04**
**Total sick leave**, median duration in days (min-max) *			
0-14 days	3 (0–14)	3 (0–14)	0.5
More than 14 days	35 (15–581)	45 (15–613)	<**0**.**001**
**Spell patterns**, median duration in days (min-max) *			
0-2 short spells	2 (0–13)	2 (0–12)	0.97
3-9 short spells	9 (3-28)	9 (3-31)	0.82
2-13 mixed spells	29 (9–421)	35 (10–321)	**0**.**02**
1-3 long spells	66 (8–581)	162 (8–613)	**0**.**002**

* All sick leave spells ended in 2005 are included, therefore maximum duration exceeds 365 days.

The majority of the eldercare workers (75%) had less than 15 sick leave days in total. Twenty-one percent had no sick leave in the study period at all. A low frequency of short-term spells, defined by zero to two spells, was present among 54% of the employees (Table 1). Homecare (28%) and non-care (26%) personnel had the highest prevalence of having more than 14 sick leave days in total compared with managers (13%) who had the lowest prevalence (Table 2). This tendency was repeated in the spell patterns; the home care personnel had the lowest prevalence of 0–2 short-term spells as well as the highest prevalence of 3–9 short-term spells and mixed spells. The prevalence of having 1–3 long-term spells was similar among the occupational groups (Table 2).

**Table 2:** Distribution of socio-demographics, categorised sick leave and number of spells in total sick leave and spell patterns

	**Total sick leave**		**Spell patterns**			
	**0**-**14 days**	**>14 days**	**0**-**2 short spells**	**3**-**9 short spells**	**2**-**13 mixed spells**	**1**-**3 long spells**
	**n (%) / mean (sd)**		**n (%)**			
	***N*** **=** ***1*,*909***	***N*** **=** ***625***	***N*** **=** ***1***,***378***	***N*** **=** ***490***	***N*** **=** ***515***	***N*** **=** ***151***
**Age**						
= < 40 years	332 (72)	130 (28)	213 (46)	118 (26)	102 (22)	29 (6)
>40 years	1,577 (76)	495 (24)	1,165 (56)	372 (18)	413 (20)	122 (6)
**Gender**						
Female	1,825 (75)	608 (25)	1,315 (54)	470 (19)	500 (21)	148 (6)
Male	84 (83)	17 (17)	63 (62)	20 (20)	15 (15)	3 (3)
**Occupation**						
Administration	50 (83)	10 (17)	44 (73)	6 (10)	6 (10)	4 (7)
Manager	177 (87)	26 (13)	160 (79)	16 (8)	17 (8)	10 (5)
Therapist/nurse	360 (82)	81 (18)	274 (62)	69 (16)	73 (17)	25 (6)
Home care	1,216 (72)	471(28)	814 (48)	382 (23)	391 (23)	100 (6)
Non-care	106 (74)	37 (26)	86 (60)	17 (12)	28 (20)	12 (8)
**Categorised sick leave**						
0 days			536 (100)	Not available	Not available	Not available
1–14 days			842 (61)	435 (32)	64 (5)	32 (2)
15–56 days			Not available	55 (13)	327 (78)	40 (10)
More than 57 days			Not available	0 (0)	124 (61)	79 (39)
**Total number of spells**	1.58 (1.5)	3.63 (1.9)				

Male workers (83%) were more likely to have 0–14 sick leave days in total than female workers (75%). Young employees were more likely to have more than 14 days of total sick leave (28%) and 3–9 short spells (26%) than older colleagues (24% and 18%; Table 2).

### Work factor scores

The scale reliabilities were; emotional demands (ά = 0.81), demands for hiding emotions (ά = 0.60), influence (ά = 0.76), meaning of work (ά = 0.70), commitment to the workplace (ά = 0.73), role conflict (ά = 0.68), and quality of leadership (ά = 0.90). Work pace was derived from a single question.

Compared with those who had less than 15 sick days, those who had more than 14 sick days were more likely to have high work demand scores, whereas those who had less than 15 sick days were more likely than the former to have high job resource factor scores (Table 3). The proportion of eldercare workers who reported their general health to be fair-poor was higher among those with more than 14 sick leave days (25%) than those with less than 15 sick leave days (9%).

**Table 3:** Mean scores of psychosocial work factors stratified on dichotomised total sick leave days and spell patterns

			**Total sick leave**			**Spell patterns**				
	**Reference mean**	**Responders having unfavourable scores**	**0**-**14 sick leave days**	**>14 sick leave days**	**p**-**value**	**0-2 short spells**	**3-9 short spells**	**2-13 mixed spells**	**1-3 long spells**	**p-value**
	**Scores (sd)**	**n (%)**	**Mean (sd)**		*	**Mean (sd)**				***
**Demand**										
Work pace #	59.5 (19)	1,440 (57)	64.1 (21)	69.4 (21)	<0.001	63.4 (21)	66.3 (21)	68.4 (21)	71.3 (18)	<0.001
Emotional #	40.7 (24)	1,689 (67)	44.8 (19)	48.0 (19)	<0.001	44.0 (20)	46.8 (18)	47.8 (18)	48.4 (21)	0.001
Hiding emotions #	50.6 (21)	503 (20)	39.6 (20)	43.4 (20)	<0.001	39.1 (19)	41.2 (21)	42.9 (19)	44.0 (22)	0.0002
**Physical work load**	missing	972 (45)	17.8 (10)	20.3 (11)	<0.001	17.0 (9)	19.9 (10)	20.3 (11)	20.0 (12)	<0.001
**Influence §**	49.8 (21)	1,246 (50)	48.4 (20)	44.1 (22)	<0.001	49.1 (20)	45.4 (20)	44.3 (21)	44.2 (23)	<0.001
**Meaning of work §**	73.8 (16)	778 (31)	77.3 (14)	75.3 (15)	0.004	77.7 (14)	75.5 (15)	75.8 (14)	76.1 (16)	0.006
**Commitment to the workplace §**	60.9 (20)	1,539 (61)	57.7 (18)	54.1 (18)	<0.001	58.7 (17)	55.3 (18)	54.1 (18)	54.6 (19)	<0.001
**Role conflict** #	42 (17)	1,320 (53)	40.8 (16)	43.8 (16)	<0.001	39.7 (17)	44.1 (15)	43.3 (15)	44.6 (15)	<0.001
**Quality of leadership §**	55.3 (21)	1,069 (44)	57.6 (22)	51.9 (25)	<0.001	58.5 (22)	55.5 (23)	52.7 (23)	48.9 (27)	<0.001
**Bullying**			n (%)		**	n (%)				****
At least from time to time	missing	333 (13)	221 (12)	112 (18)	<0.001	145 (11)	72 (15)	85 (17)	31 (21)	<0.001
**Threats of violence**										
At least from time to time	missing	985 (39)	721 (38)	264 (43)	0.045	486 (36)	217 (45)	218 (43)	64 (43)	0.001
**General health**										
Fair-poor	missing	333 (13)	180 (9)	153 (25)	<0.001	112 (8)	68 (14)	107 (21)	46 (31)	<0.001

# (> reference mean scores) and § ( = < reference mean scores).

* t-test / **chi2.

*** one-way ANOVA / **** chi2.

The same trend as that described for total sick leave days was evident for the spell patterns; few and short-term spells had the most favourable mean work factor scores; and the scores turned more unfavourable the more frequent and/or the longer the duration of the spells (Table 3). The number of employees being bullied threatened or having a poor health increased as the frequency and the duration of the spell patterns rose.

### Total sick leave days

We estimated the odds of having unfavourable work factor scores among eldercare workers with more than 14 sick leave days compared with 0–14 days (Table 4). In crude analyses, the odds ratios for all psychosocial work environment factors were statistically significantly different from one. Adjustments for age, gender, occupation and number of spells attenuated in all of the associations, but the odds remained statistically significant for having unfavourable scores in work pace 1.41 (95% CI: 1.1 - 1.7), demands for hiding emotions 1.56 (95% CI: 1.2 - 2.0), quality of leadership 1.41 (95% CI: 1.1 - 1.7) and being bullied from time to time 1.50 (95% CI: 1.1 - 2.0).

**Table 4:** The odds of having unfavourable psychosocial work factor scores among eldercare workers

	**More than 14 sick leave days,**	**More than 14 sick leave days**
	**OR (95% CI)**	adjusted*, OR (95% CI)
**Demand**		
Work pace	1.48 (1.2 - 1.8)	1.41 (1.1 - 1.7)
Emotional	1.19 (1.0 - 1.4)	1.08 (0.9 - 1.4)
Hiding emotions	1.65 (1.3 - 2.0)	1.56 (1.2 - 2.0)
**Physical work load**	1.34 (1.1 - 1.6)	1.15 (0.9 - 1.4)
**Influence**	1.36 (1.1 - 1.6)	1.13 (0.9 - 1.4)
**Meaning of work**	1.32 (1.1 - 1.6)	1.14 (0.9 - 1.4)
**Commitment to the workplace**	1.49 (1.2 - 1.8)	1.23 (1.0 - 1.5)
**Role conflict**	1.31 (1.1 - 1.6)	1.11 (0.9 - 1.4)
**Quality of leadership**	1.50 (1.2 - 1.8)	1.41 (1.1 - 1.7)
**Bullying**		
At least from time to time	1.68 (1.3 - 2.2)	1.50 (1.1 - 2.0)
**Threats of violence**		
At least from time to time	1.21 (1.0 - 1.5)	1.03 (0.8 - 1.3)

The odds of having unfavourable psychosocial work factor scores with more than 14 sick leave days compared with 0-14 days.

* Adjusted for age, gender, occupation and number of spells.

### 0–2 short-term spells compared with any other spell pattern

Crude analyses showed that the odds for having unfavourable work factor scores were significantly higher for employees with spell patterns featuring 3–9 short spells or 2–13 mixed spells than for employees with 0–2 short-term spells (Table 5). However, according to the crude analyses, employees having 1–3 long-term spells did not give statistically significantly more unfavourable scores to emotional demands, physical work load, influence, meaning of work or role conflict than employees with 0–2 short-term spells. After adjustment for age, gender, occupation and total sick leave days, employees having 3–9 short-term spells had significantly increased odds of unfavourable scores for role conflict 1.50 (95% CI: 1.2-1.9) than employees with 0–2 short-term spells. For the mixed spell pattern, the odds of having unfavourable work factor scores compared with 0–2 short-term spells were highest in emotional demands 1.62 (95% CI: 1.1-2.5) after adjustment. Work pace 2.24 (95% CI: 1.4-3.7) was more strongly associated with 1–3 long spells than with 3–9 short-term spells and 2–13 mixed spells.

**Table 5:** The odds of having unfavourable psychosocial work factor / general health scores among eldercare workers

	**3**-**9 short spells**	**3**-**9 short spells**	**2**-**13 mixed spells**	**2**-**13 mixed spells**	**1**-**3 long spells**	**1**-**3 long spells**	
	**OR (95% CI)**	OR (95% CI) *	**OR (95% CI)**	OR (95% CI) *	**OR (95% CI)**	OR (95% CI) *	**p**-**value #**
**Demand**							
Work pace	1.26 (1.0 -1.6)	1.28 (1.0 - 1.6)	1.51 (1.2 - 1.9)	1.52 (1.0 - 2.2)	2.10 (1.4 - 3.0)	2.24 (1.4 - 3.7)	**0**.**01**
Eemotional	1.32 (1.1 - 1.7)	1.32 (1.0 - 1.7)	1.40 (1.1 - 1.7)	1.62 (1.1 - 2.5)	1.27 (0.9 - 1.8)	1.37 (0.8 - 2.3)	0.07
Hhiding emotions	1.40 (1.1 - 1.8)	1.21 (0.9 - 1.6)	1.55 (1.2 - 1.20)	0.97 (0.6 - 1.5)	2.15 (1.5 - 3.1)	1.42 (0.8 - 2.4)	0.19
**Physical work load**	1.52 (1.2 - 1.9)	1.29 (1.0 - 1.7)	1.51 (1.2 - 1.9)	1.20 (0.8 - 1.8)	1.36 (0.9 - 2.0)	1.16 (0.7 - 1.9)	0.26
**Influence**	1.28 (1.0 - 1.6)	1.01 (0.8 - 1.3)	1.51 (1.2 - 1.8)	1.19 (0.8 - 1.7)	1.29 (0.9 - 1.8)	1.13 (0.7 - 1.8)	0.83
**Meaning of work**	1.33 (1.1 - 1.7)	1.11 (0.9 - 1.4)	1.43 (1.2 - 1.8)	1.03 (0.7 - 1.5)	1.14 (0.8 - 1.6)	0.81 (0.5 - 1.3)	0.54
**Commitment to the workplace**	1.46 (1.2 - 1.8)	1.17 (0.9 - 1.5)	1.67 (1.3 - 2.1)	1.28 (0.9 - 1.9)	1.50 (1.0 - 2.1)	1.19 (0.7 - 1.9)	0.52
**Role conflict**	1.54 (1.2 - 1.9)	1.50 (1.2 - 1.9)	1.43 (1.2 - 1.7)	1.25 (0.9 - 1.8)	1.25 (0.9 - 1.8)	1.05 (0.7 - 1.7)	**0**.**01**
**Quality of leadership**	1.27 (1.0 - 1.6)	1.17 (0.9 - 1.5)	1.56 (1.3 - 1.9)	1.38 (0.9 - 2.0)	1.56 (1.3 - 1.9)	1.77 (1.1 - 2.9)	0.11
**Bullying**							
Aat least from time to time	1.46 (1.1 - 2.0)	1.29 (0.9 - 1.8)	1.68 (1.3 - 2.2)	1.14 (0.7 - 2.0)	2.27 (1.5 - 3.5)	1.39 (0.7 - 2.6)	0.41
**Threats of violence**							
Aat least from time to time	1.45 (1.2 - 1.8)	1.17 (0.9 - 1.5)	1.34 (1.1 - 1.7)	1.19 (0.8 - 1.8)	1.37 (1.0 - 1.9)	1.28 (0.8 - 2.1)	0.55
**General health**							
Ffair-poor	1.84 (1.3 - 2.5)	1.56 (1.1 - 2.3)	2.98 (2.2 - 4.0)	1.29 (0.7 - 2.2)	4.97 (3.3 - 7.4)	2.10 (1.1 - 3.9)	**0**.**01**

The odds of having unfavourable psychosocial work factor/general health scores with different sick leave patterns compared with 0-2 short spells.

* Adjusted for age, gender, occupation, total sick leave days # Wald-test for overall difference in work factor score bestween sick leave patterns.

The odds of having a fair-poor general health were most strongly associated with 1–3 long-term spells; even after adjustments, the odds ratio was statistically significantly different from one; 2.10 (95% CI: 1.1-3.9).

The Wald-test showed an overall statistically significant difference in the odds of having unfavourable scores in work pace, role conflict and general health between the sick leave patterns (Table 5).

### Effect modification by age

Age significantly modified the association between a frequent short-term sick leave pattern and commitment to the workplace and between frequent short-term sick leave and quality of leadership (Table 6). For those below 40 years of age, the odds ratio of having unfavourable scores in commitment to the workplace was 1.33 (95% CI: 0.8 - 2.2) when having a frequent spell pattern compared with 1–3 long spells, whereas older colleagues had an odds ratio of 0.14 (95% CI: 0.03 - 0.6). Among young employees, the odds ratio of having unfavourable scores in quality of leadership was 0.80 (95% CI: 0.5 - 1.3) among frequent absentees compared with those having 1–3 long spells. Among older employees, this odds ratio was 0.30 (95% CI: 0.1 - 0.7). No statistically significant effect modification was seen in relation to general health.

**Table 6:** The modifying effect of age on associations between unfavourable psychosocial work factor / general health scores and spell pattern

	**3-9 short spells**	**Interaction term**		
	OR (95% CI) *	**p-value**	**Old age (>40)**	**Young age (= < 40)**
**Demand**				
Work pace	0.57 (0.3 - 0.9)	0.28	0.86 (0.4 - 2.1)	0.51 (0.3 - 0.9)
Emotional	0.97 (0.6 - 1.6)	0.75	0.87 (0.3 - 2.2)	1.02 (0.6 - 1.8)
Hiding emotions	0.85 (0.5 - 1.4)	0.84	0.78 (0.3 - 2.3)	0.87 (0.5 - 1.5)
**Physical work load**	1.11 (0.7 - 1.8)	0.85	1.22 (0.5 - 3.1)	1.11 (0.6 - 1.9)
**Influence**	0.90 (0.6 - 1.4)	0.22	0.56 (0.2 - 1.4)	1.01 (0.6 - 1.7)
**Meaning of work**	1.37 (0.8 - 2.2)	0.11	0.74 (0.3 - 1.8)	1.63 (0.9 - 2.8)
**Commitment to the workplace**	0.98 (0.6 - 1.6)	**0**.**004**	0.14 (0.03 - 0.6)	1.33 (0.8 - 2.2)
**Role conflict**	1.42 (0.9 - 2.3)	0.06	0.66 (0.3 - 1.7)	1.69 (1.0 - 2.8)
**Quality of leadership**	0.66 (0.4 - 1.1)	**0**.**05**	0.30 (0.1 - 0.7)	0.80 (0.5 - 1.3)
**Bullying**				
At least from time to time	0.93 (0.5 - 1.7)	0.66	1.17 (0.4 - 3.8)	0.89 (0.5 - 1.7)
**Threats of violence**				
At least from time to time	0.91 (0.6 - 1.5)	0.55	1.15 (0.5 - 2.8)	0.86 (0.5 - 1.4)
**General health**				
Fair-poor	0.74 (0.4 - 1.3)	0.22	0.41 (0.1 - 1.2)	0.83 (0.5 - 1.5)

The modifying effect of age on the association between unfavourable work factor / general health scores and 3-9 short spells compared with 1-3 long spells.

* Adjusted for age, gender, occupation, total sick leave days.

## Discussion

Signs of a poor working environment reflected in unfavourable job demand and job resource scores were consistently, significantly associated with employees having more than 14 sick leave days compared with those having less than 15 days. For unfavourable scores in work pace, demands for hiding emotions and being bullied, at least from time to time, as well as quality of leadership, this association remained present among employees with more than 14 sick leave days after adjustment for the number of sick leave spells.

After adjustments for the effect of sick leave length, unfavourable scores in role conflict and emotional demands remained associated with a frequent spell pattern, i.e. 3–9 short-term spells and 2–13 mixed spells, respectively, compared with a non-frequent short-term spell pattern. The strongest association with the long-term spell pattern was seen among employees with unfavourable work pace scores.

Our results neither confirmed nor denied the hypothesis that a frequent short-term sick leave spell pattern was more strongly associated with an unfavourable working environment than a long-term sick leave spell pattern after adjustment for the effect of total sick leave length.

The hypothesis about general health was supported by our results; hence, a long-term sick leave spell pattern was more likely to be associated with poor general health than a short-term sick leave spell pattern.

Age significantly modified the association between sick leave patterns and work factors in the hypothesised direction for commitment to the workplace. Compared with a long-term sick leave pattern, a frequent short-term sick leave pattern decreased the odds ratio of having unfavourable scores in commitment to the workplace among older employees as opposed to among the young employees whose odds ratio of having unfavourable work environment factors increased.

### Other studies

#### Sick leave length

In the perspective of the job demands-resources model; long-term sick leave, irrespectively of spell frequency, is more associated to high levels of demanding work factors than short-term sick leave [14]. In the survey conducted by NRCWE (10;11) the identified work factors associated with self-reported sick leave length may inform about the most relevant demands in the eldercare sector. Crude results from our analyses of the association between sick leave length and work factors were in accordance with those found by Borg et al. [10]. After adjustment for age, gender, occupation and number of spells, associations were attenuated. Work pace, demands for hiding emotions and bullying remained significant associated with sick leave length irrespectively of frequency of spells and may be unique demanding factors in this particular work setting. No previous studies analysing possible associations between sick leave length and work environment factors in the healthcare sector [15,16,23] have adjusted for the number of spells. However, high psychological demands and high work load [15] and job demands [16] defined by the job demand-control-support model by Karasek were associated with sick leave duration. The study by Schreuder et al. found that the demand-control ratio (high job demands and low control) was inversely associated with the number of sick leave days [23]. This is a surprising result but it may underline that the items in the demand-control-support model may not capture the uniqueness of the healthcare work environment [7].

#### Frequency of sick leave spells

A direct comparison between our study and studies also reporting short-term sick leave spells was possible. A high frequency of 1–7 days spells was associated with low levels of respect from a supervisor [23]. Also, a low decision latitude and a low predictability at work increased the risk of having a frequent 1–10 days spell pattern among women [17]; on the other hand, we saw a tendency towards demanding work factors being more likely to be associated with 3–9 short-term sick leave spells or mixed spells than 0–2 short-term spells. No such association was found for resources in the work environment.

High mean scores in emotional demands and role conflict were the only work factors that remained statistically significantly associated with having a frequent spell pattern after adjustment for total sick leave. This may imply that among employees in the eldercare sector, demanding work factors in general and emotional demands and role conflict in particular are more associated with frequency of sick leave spells than available resources at work. It contradicts the job demands-resources model which claims that frequent sick leave spells, regardless of the sick leave length, are likely to be more associated to low levels of motivational work factors than non-frequent spells [14]. Low levels of role conflict may however, be viewed upon as motivational; it is one of the scales about “interpersonal relations and leadership” in COPSOQ which also contains scales about motivational factors like feed back at work, and social support [32] referred to as job resources in the study of Schaufeli et al. [14].

Some studies have also used a set of variables to describe the working environment [19], or they have used the demand-control-support model by Karasek and Theorell and the effort-reward model by Siegrist [23]. They found that a high number of one-day spells was associated with low support, low influence and high physical load [19]. They also found that frequent 1–7 days absentees were more likely to display high levels of effort and to get low rewards in return, but also that low job demands and high control were associated with that sick leave pattern [23]. We analysed all work factors separately. However, a post-analysis of the linear effect of the number of work factors perceived as stressful showed that the number of stressful work factors was increased (β = 0.54; 95% CI: 0.3-0.8) among those with 3–9 short-term sick spells compared with those with 0–2 short-term spells after adjustment for age, gender and occupation. This is equivalent to an increase in odds ratio from 1.1 (95% CI: 1.05-1.1) to 1.5 (95% CI: 1.2-1.7) when the number of stressful work factors rose from one to four. This is similar to the results of Elstad et al.; they reported that the odds ratio rose from 1.3 to 1.5 when the number of items perceived as stressful rose from one to four among Nordic eldercare workers [20]. However, these results were based on spells of any duration, whereas our results pertain exclusively to short-term spells.

We found that long-term sick leave spells were significantly associated with unfavourable scores in work pace, demands for hiding emotions, quality of leadership and being bullied. These findings correspond nicely with those of other studies; a higher probability of having long-term sick leave spells was associated with high psychological job demands [17], physical demands, mental demands and bullying [18], and quality of leadership [22]. Their definition of long-term sick leave spells (more than 10 days, more than 27 days and, finally, more than 8 weeks) was, however, different from the one used in the present study (more than 7 days). Redefining long-term sick leave spells to 15–56 days or more than 56 days in our data strengthened the associations, but the confidence intervals became much wider (results not shown).

This strengthening effect may seem surprising because sick leave spells of a long duration are viewed upon as health-related and not as a strategy deployed to cope with an unfavourable psychosocial work environment [12-14]. Employees in the eldercare sector have high levels of sickness presenteeism, i.e. going to work despite ill health [20]. Commitment to the elderly and their colleagues probably explains this phenomenon; exposed to further stress at work has been shown to increase the number of episodes of presenteeism; and the risk of future sick leave may well increase with more presenteeism episodes [20]. Our results possibly reflect the long-term effects of being exposed to unfavourable work factors and repeated presenteeism episodes causing prolonged sick leave.

We think of general health as an intermediate factor in the association between sick leave and unfavourable work factor scores. Long term sick leave is found to be predicted by the exposure of work demands in the study by Schaufeli et al. although it is recognised that health status may as well influence the long-term sick leave [14]. We therefore analysed self-rated general health as a dependent variable. Expectedly, it was clearly associated more with a long-term sick leave spell pattern than with a short-term and mixed spell pattern (12;13). We also analysed whether general health modified the association between spell patterns and the working environment and it did not (results not shown). This implies that the use of sick leave patterns as an indicator of unfavourable work factors is equally good/bad, i.e. it is independent of the respondent’s general health status.

#### Interaction between age and spell patterns

We found that a statistically significantly higher proportion of young employees (26%) than older employees (18%) had 3–9 short-term sick leave spells. This result is in accordance with results reported by other studies [17,19,20,23,24]. Our data, however, did not confirm the often made observation that the proportion of employees who have few, but long sick leave spells is higher among older than among young employees [17,18,24]. In our material, about 6% had 1–3 long-term spells both among young and older eldercare workers.

We identified no studies that analysed whether age acted as an effect modifier in the association between sick leave pattern and work factors among health care employees. One study by Donders et al. investigated the moderating effect of age between work-related characteristics and frequent sick leave and prolonged sick leave among employees at a Dutch university [25]. They found that among employees younger than 46 years the OR of having three or more sick leave spells was decreased when decision latitude was high. This was not significant among the older colleagues. Borg et al. concluded that the increased sick leave levels among young eldercare workers were explained mainly by more role conflict and less commitment to the work place [11]. We wanted to explore whether this observation was linked to the duration or the frequency of the spells rather than overall sick leave length. Our results showed that an unfavourable score in commitment to the workplace was associated with a frequent short-term sick leave spell pattern as opposed to a long-term pattern among young employees. The opposite effect was seen among older colleagues. We found no statistically significantly effect modification in relation to role conflict. Unfavourable scores in quality of leadership were associated more with a long-term sick leave spell pattern than with a frequent short-term spell pattern; and this association was significantly stronger among old than among young employees. In the study by Donders et al.; the OR for frequent sick leave decreased when young employees scored high on decision latitude, whereas the OR became insignificant among older colleagues. High scores in physical workload and conflict with superiors increased the OR for prolonged sick leave among all age categories [25].

One possible explanation for this difference in similar study populations may be rooted in differences between the data sources. Our sick leave data are register-based as opposed to the self-reported sick leave length used in previous studies. We have previously shown that the precision in self-reported sick leave among young (19–29 years of age) eldercare workers was lower than the precision of register data and that eldercare workers were poor at recalling their sick leave and mostly under-reported the actual length by more than seven days [34].

### Strengths and limitations

We have contributed with new insight into how the frequency of sick leave spells interacts with sick leave length and how sick leave spells are associated with work factors and general health in the municipal eldercare sector.

It strengthened the validity of our results that the sick leave measures were constructed from register-based sick leave data. This reduced the risk of making trivial associations [35].

We excluded 779 employees because they were not employed throughout the entire year of 2005. By that we missed 40 responders to the questionnaire about the working environment. They were younger (median age 40.1 years) than the study population, and 85% were occupied in home care. This might have caused selection bias toward the null hypothesis because their mean work factor scores were neither more nor less favourable.

Non-responders (24%) were more likely to be young, having more than 14 sick leave days in total and having more long-term sick leave spells than responders. This might have weakened the association between sick leave length / long-term sick leave spell pattern and work factors due to selection bias. Also, a possible underestimation of the association between spell frequency and work factors adjusted for sick leave length cannot be ruled out.

Two of the work factor scales; demands for hiding emotions (ά = 0.60) and role conflict (ά = 0.68) showed lower alphas than the 0.7-threshold that is widely recognised [36]. In the development of first version of COPSOQ [32] the psychometric properties of demands for hiding emotions (ά = 0.59) were also low. This scale is short and consist of two items, whereby the probability of getting an alpha lower than 0.7 is increased [32]. Role conflict, on the other hand consist of four items and showed a higher alpha (ά = 0.72) in the original version [32] than in this present study. Using a valid yet unreliable scale as the dependent variable statistical power may decrease [37]. However, it has been shown that Cronbach’s alpha may underestimate the true reliability when combined items in a scale have common causes rather than common effects. Higher intra class coefficients retest reliabilities for demands for hiding emotions (0.75) and role conflict (0.74) were found than if reliabilities were expressed by alphas [37].

We studied demands and resources found in a representative sample of Danish eldercare employees’ to be associated with self-reported sick leave length (10;11). We have no evidence, though that these work factors uniquely describe demands and resources of the eldercare sector from a theoretical point of view. Future research should focus on whether information about sick leave patterns may be used to enhance interventions that aim at improving the work environment in this particular work setting.

## Conclusions

Sick leave length was a better indicator of unfavourable scores in work pace, demands for hiding emotions, quality of leadership and bullying than a sick leave length below 15 days and a 0–2 short-term sick leave spell pattern, respectively. A frequent short-term sick leave spell pattern was a better indicator of unfavourable scores in emotional demands and role conflict than overall sick leave length. General health was strongly associated with the long-term sick leave pattern.

The present study could not confirm the hypothesis that an association between unfavourable work factor scores and total sick leave length was more likely to be due to the frequency of spells than their duration and that this association would be more pronounced among young employees than among their older colleagues. However, scores in commitment to the workplace and quality of leadership varied with sick leave pattern and age. These conclusions underpin that different sick leave measures may be associated with different work environment factors. Further studies on these associations may inform interventions to improve occupational health care.

## Competing interests

The authors declare that they have no competing interests.

## Authors’ contributions

CMS conceived the study, carried out statistical analyses and drafted the manuscript. NTA supervised the statistical analyses. All authors participated in the design of the study, helped to draft the manuscript and interpreted the results. All authors have read and approved the final manuscript.

## Pre-publication history

The pre-publication history for this paper can be accessed here:

http://www.biomedcentral.com/1471-2458/13/578/prepub
